# BCGLMs: Bayesian modeling for disease prediction using compositional microbiome features

**DOI:** 10.1093/bioadv/vbag041

**Published:** 2026-02-11

**Authors:** Li Zhang, Zhenying Ding, Nengjun Yi

**Affiliations:** Biostatistics and Bioinformatics Facility, Fox Chase Cancer Center, Philadelphia, PA 19111, United States; Department of Biostatistics, University of Alabama at Birmingham, Birmingham, AL 35294, United States; Department of Biostatistics, University of Alabama at Birmingham, Birmingham, AL 35294, United States

## Abstract

**Motivation:**

**BCGLMs** is a freely available R package that provides functions for setting up and fitting Bayesian compositional models for continuous, binary, ordinal and survival responses. It also includes models with random effects to capture sample-related accumulated small effects, improving prediction accuracy. The package includes tools for summarizing results from fitted models both numerically and graphically. Built on top of the widely used **brms** package, **BCGLMs** enable users to incorporate phylogenetic relationships between microbiome taxa into the modeling framework. Overall, **BCGLMs** package offers a flexible and powerful set of tools for analyzing compositional microbiome data.

**Availability and implementation:**

The package is publicly available via GitHub https://github.com/Li-Zhang28/BCGLMs.

## 1 Introduction

Recent advances in sequencing technologies and computational tools—such as 16S rRNA gene sequencing and taxonomic profiling—have enabled high-throughput characterization of the human microbiome (Jovel *et al.* 2016, Johnson *et al.* 2019). These developments have led to the generation of microbiome data summarized as operational taxonomic units (OTUs) (Huse *et al.* 2012). It facilities researchers exploring the relationships between microbiome composition and health outcomes, with growing interest in using microbiome features to predict disease and inform personalized medicine strategies.

However, incorporating microbiome data as covariates in predictive models presents several analytical challenges: (i) microbiome data are compositional, meaning only relative abundances are observed (Gloor *et al.* 2017). Due to high sparsity and variability in count data, these covariates are typically transformed and analyzed using compositional data analysis techniques; (ii) the hierarchical and correlated structure of microbiome data, embedded within a phylogenetic tree and classified across taxonomic levels, introduces intrinsic relationships among taxa that complicate variable selection and coefficient estimation (Martiny *et al.* 2015); (iii) the high dimensionality of microbiome data—often involving thousands to tens of thousands of taxa—contrasts sharply with the relatively small number of available samples, necessitating advanced regularization or Bayesian shrinkage approaches (Chen *et al.* 2013); (iv) modeling disease outcomes is complicated by their diverse forms, including continuous measures, binary indicators, ordinal stages, and time-to-event outcomes, requiring flexible modeling frameworks (Zhang *et al.* 2024a,b).

Various methods have been proposed to address these challenges. To handle the high dimensionality of microbial features, researchers have employed Bayesian approaches and penalized regression techniques. The compositional nature of microbiome data—has typically been addressed through sum-to-zero constraints on the coefficients (Lin *et al.* 2014, Shi *et al.* 2016). To account for the phylogenetic and taxonomic relationships among microbial taxa, structured Bayesian priors have also been developed (Zhang *et al.* 2021, 2024a,b). While initial methods focused on continuous outcomes, some have been extended to binary responses (Lu *et al.* 2019). In our recent work (Zhang *et al.* 2024a), we further advanced this line of research by developing models capable of handling ordinal outcomes through a Bayesian framework using Markov Chain Monte Carlo (MCMC) algorithms.

Despite these methodological developments, few R packages are currently available to implement such models for compositional microbiome data. The **Compack** package implements the penalized log-contrast model proposed by Lin *et al.* (2014) for continuous outcomes. The **coda4microbiome** package, developed by Calle and Susin, applies elastic-net penalization to all possible pairwise log-ratios in generalized linear models (GLMs), supporting continuous and binary outcomes(Calle and Susin 2022). Additionally, the *bglm* function in the **BhGLM** package enables Bayesian hierarchical modeling, including applications to compositional data(Yi *et al.* 2019, Zhang *et al.* 2025a).

However, these packages are limited in several important ways. All are restricted to either binary or continuous outcomes, with **Compack** only applicable to continuous data. Moreover, both **Compack** and **coda4microbiome** rely on penalization-based regression approaches that yield point estimates and do not provide full posterior distributions, thus failing to capture uncertainty in parameter estimates and predictions. Crucially, none of these packages incorporate phylogenetic relationships among microbial taxa, which are essential for accurately modeling the inherent structure of microbiome data.

A flexible and efficient R package is essential for advancing the analysis of compositional microbiome data in disease research. We present **BCGLMs**, a freely available R package designed to predict disease outcomes using compositional microbiome predictors, based on our previously published methods. The package implements four Bayesian modeling approaches: BCGLM, BCO, BCCOXPH, and BCGLMM. In our prior work, we evaluated against existing tools and demonstrated that BCGLM outperforms the popular functions in packages such as **Compack** and **coda4microbiome**, yielding more accurate coefficient estimates and lower prediction error in compositional data analysis ([Bibr vbag041-B20][Bibr vbag041-B21]). The BCO method is the first and only approach developed to handle multi-level ordinal responses using compositional microbiome data (Zhang *et al.* 2024a). The BCGLMM method introduces additional innovation by incorporating random effects, allowing for the detection of both moderate effects from specific taxa and the cumulative influence of many low-abundance taxa ([Bibr vbag041-B18][Bibr vbag041-B19]). Together, these tools make **BCGLMs** a comprehensive and practical solution for microbiome-based disease modeling.

## 2 Models and algorithms

Assume we collected n microbiome samples. For each sample, we observe a response variable y and *m* compositional microbiome taxa, represented as relative abundance tax—i.e. the observed counts divided by the total sequences. Let ${X=(x}_{ij})$ denote the resulting n×m matrix of microbiome covariates, with ($x_{ij}\geq 0$) and $\sum_{j=1}^{m}x_{ij}=1$ for all *i*. The response y may be continuous, binary, ordinal or survival. We link the response to the microbiome covariates, using an appropriate link function depending on the response type. To account for the compositional nature of the microbiome covariates, we use log-contrast models with sum-to-zero constraint (Lin *et al.* 2014, Shi *et al.* 2016, Lu *et al.* 2019):


$$\eta =\log\;(X)\beta ,\;\sum_{j=1}^{m}\beta_{j}=0$$

In generalized linear models, η is linked to the response *y* through a link function h(·):


$$\mu_{i}=E(y_{i}|\eta_{i})=h^{-1}(\eta_{i})$$

For survival outcome, we consider the Cox proportional hazards model, where the hazard function of the survival time t is modeled as:


$$h(t|\eta_{i})=h_{0}(t)\;*\;\mathrm{ex}p\left(\eta_{i}\right),\;\sum_{j=1}^{m}\beta_{j}=0$$

This sum-to-zero constraint ensures identifiability and reflects the compositional nature of the data. In Bayesian framework, this constraint can be softly enforced by assigning a prior (Zhang *et al.* 2024a,b, 2025a,b):


$$\sum_{j=1}^{m}\beta_{j}∼N(0,0.001\;*\;m).$$

which we refer to as “soft-centering”. This approach allows for implementation within the **brms** package in R, which provides a flexible interface for Bayesian modeling using **Stan** (Gelman *et al.* 2015). The **brms** framework enables user-defined priors and automatically compiles the corresponding **Stan** code, facilitating model specification and estimation (Bürkner 2017).

To account for phylogenetic and taxonomic relationships between taxa and incorporate this relation in variable coefficients estimation, we use regularized horseshoe priors on compositional covariates coefficients(Piironen and Vehtari 2017):


$$\begin{array}{cc} & \beta_{j}|\lambda_{j},\tau ,c∼N(0,\tau^{2}{{\tilde{\lambda }}_{j}}^{2}),\\ & {{\tilde{\lambda }}_{j}}^{2}=\frac{c^{2}\lambda_{j}^{2}}{c^{2}+\tau^{2}\lambda_{j}^{2}}\\ & \lambda_{j}∼\mathrm{half}-\mathrm{Cauchy}(0,1)\\ & \tau ∼\mathrm{half}-\mathrm{Cauchy}(0,\tau_{0}^{2})\\ & c^{2}∼\mathrm{Inv}-\mathrm{Gamma}(\nu /2,{\nu s}^{2}/2) \end{array}$$

the regularized horseshoe is a continuous shrinkage prior that includes both a global scale parameter τ, and the local shrinkage parameters $\lambda_{j}$ for each $\beta_{j}$. The global parameter τ controls overall shrinkage across all taxa, while the local parameters $\lambda_{j}$ allow for coefficient-specific regularization. This structure enables the model to apply strong shrinkage to irrelevant taxa while preserving signals with substantial effects, thereby improving estimation accuracy in high-dimensional settings.

We incorporate phylogenetic structure by modeling the local shrinkage parameters $\lambda_{j}$ based on a taxon-level similarity matrix W (Randolph *et al.* 2018), constructed from a phylogenetic square distance matrix *D*^(2)^ (Randolph *et al.* 2018):


$$W=-\frac{1}{2}(I-\frac{1}{m}11^{T})D^{\left(2\right)}(I-\frac{1}{m}11^{T})$$

This structure is modeled using an intrinsic autoregressive (IAR) prior (Morris *et al.* 2019), which induces correlated shrinkage across phylogenetically related taxa. This hierarchical structure can also be implemented via **brms** through custom prior specification.

In the BCGLMM model, we further introduce a sample-level random effect u to capture the cumulative small effects ([Bibr vbag041-B18][Bibr vbag041-B19]),


$$\begin{array}{l} \eta_{i}=\beta_{0}+\log\;(X_{i})\beta +u_{i}\\ u∼\mathrm{MV}N_{n}(0,Kv) \end{array}$$

where K is a sample-level similarity matrix constructed to encode relationships among samples, such as shared environmental or clinical features (Zhao *et al.* 2015).

The model is estimated using the Hamiltonian Monte Carlo (HMC) algorithm and its adaptive variant, the No-U-Turn Sampler (NUTS), in **Stan** (Gelman *et al.* 2014). These MCMC methods efficiently generate posterior samples from the joint distribution defined by the likelihood and the prior specifications. The resulting posterior draws are used to summarize the distribution of each model parameter, enabling both point and interval estimation, and supporting inference on effect sizes and prediction accuracy.

## 3 Features

In the developed R package, we provide four core functions: *bcglm*, *bco*, *bccoxph*, and *bcglmm*, which implement compositional generalized linear models (GLMs), compositional cumulative logistic regression, compositional survival CoxPH regression, and compositional GLM mixed models, respectively. For *bcglm*, *bco*, and *bccoxph*, incorporating a similarity matrix between taxa is optional. To facilitate this, we include a *similarity* function that computes the similarity matrix based on phylogenetic or taxonomic information. Users can choose whether to include this matrix in the model; by default, the similarity matrix is not used, meaning the phylogenetic relationships among taxa are ignored.

To support model implementation, the package also provides simulation functions for each model type. The functions *sim_c* and *sim_b* generate simulated microbiome covariates under a compositional framework for continuous and binary response, respectively, as described in our paper ([Bibr vbag041-B20][Bibr vbag041-B21]). The *sim_s* function generates simulated compositional variables for survival responses. The *sim_o* function generates simulated compositional variables for ordinal responses (Zhang *et al.* 2024a), and *sim_cmm* simulates compositional variables with a mixture of both large and small effect sizes, where the proportion of small effects can be specified relative to the total number of predictors ([Bibr vbag041-B18][Bibr vbag041-B19]).

The **BCGLMs** package uses **brms** as the underlying Bayesian regression engine for model fitting. Therefore, summary and diagnostic tools provided by **brms**—such as *summary()*, *fixef()*, and *plot()*—remain fully available to users. These functions allow users to extract posterior summaries (e.g. means, standard errors, and 95% credible intervals for regression coefficients) and to generate standard diagnostic figures, including trace plots and posterior density plots. The *mcmc_plot()* function, implemented in **bayesplot** and accessible through **brms**, provides additional visualization of posterior estimates and uncertainty.

## 4 Results

In our published work introducing the bcglm, bco, and bcglmm methods, we conducted simulation studies to evaluate the performance of each approach. In this section, we demonstrate how to apply each method using the corresponding simulation functions provided in the package. These functions ultimately call the *brm* function from the **brms** package, with the regularized horseshoe prior incorporated for coefficient estimation. In the work (Zhang *et al.* 2024a,b), we have shown that the global shrinkage parameter τ with a heavy-tailed Cauchy prior is minimally impacted by the choice of the scale $\tau_{0}$; in this article, we use the default setting with a scale of 1.

To use the **BCGLMs** package, users can install it from GitHub. The package depends on several R packages, including **phyloseq**, **BhGLM**, and **brms**. For users running R version 4.4.x, the following code can be used to install the required packages and BCGLMs:

#Install dependency

if (! require(“BiocManager”, quietly = TRUE)) {

 install.packages(“BiocManager”)

}

BiocManager::install(“phyloseq”)

install.packages(“remotes”)

remotes::install_github(“paul-buerkner/brms”)

remotes::install_github(“nyiuab/BhGLM”, force = TRUE)

Note:

Please check Bioconductor—phyloseq for more versions of phyloseq.

BhGLM is currently compatible with *R* ≤ 4.4.x. Users running *R* ≥ 4.5 may encounter installation or runtime errors because the package has not been updated for recent R versions.

#Install BCGLMs

remotes::install_github(“Li-Zhang28/BCGLMs”, force = TRUE, build_vignettes = TRUE)

Here, we demonstrate the use of ***bcglm*** to analyze example data.

### 4.1 Demonstration of *bcglm* using simulated data

The *bcglm* function is designed to analyze compositional microbiome data with either continuous or binary outcomes. The simulation functions *sim_c* and *sim_b* generate correlated compositional data with continuous and binary responses, respectively, replicating the setup from our paper ([Bibr vbag041-B20][Bibr vbag041-B21]). Users can specify the sample size and number of taxa (i.e. covariate dimensionality), and the output is a list containing simulated compositional predictors and outcomes.

After generating the data, users can fit the model using *bcglm*, with the option to include a taxon-level similarity matrix. We also provide a *similarity* function to compute this matrix based on phylogenetic or taxonomic information. The inclusion of this similarity structure allows the model to account for phylogenetic relationships among taxa during estimation.

In this simulation, we generated 100 compositional variables and a continuous response for 400 samples. The similarity matrix was calculated and stored as sim, which was then incorporated into the model fitting using the *bcglm* function. The parameters df_local and df_global specify the degrees of freedom for the Student-*t* distributions on the prior of local shrinkage parameters $\lambda_{j}$ and the global shrinkage parameter τ in the regularized horseshoe prior. By default, both parameters are set to 1, corresponding to a half-Cauchy distribution. Users may also define half-*t* priors with smaller degrees of freedom to induce heavier tails and allow for more flexible shrinkage behavior.

dat = sim_c( *n* = 400,  *p* = 100)

otu=otu_table(dat$x, taxa_are_rows = F)

dis.taxa = phyloseq::distance(otu, method = “bray”, type = “taxa”)

sim = similarity(dis.taxa)

fit = bcglm(x = dat$x, y = dat$y, family = gaussian, df_local = 1, df_global = 1, similarity = sim)

After model fitting, posterior summaries—including the point estimates and the 2.5% and 97.5% quantiles—can be extracted for the regression coefficients.

**Table:** 

> fixef(fit)[1:10 , ]				
	Estimate	Est. Error	Q2.5	Q97.5
Intercept	−0.1966	0.5505	−1.2855	0.8732
X1	−0.0024	0.0390	−0.0952	0.0789
X2	0.0019	0.0390	−0.0806	0.0910
X3	−0.0030	0.0381	−0.0936	0.0778
X4	−0.0380	0.0587	−0.1897	0.0404
X5	−0.0237	0.0497	−0.1553	0.0486
X6	−0.0482	0.0648	−0.2131	0.0332
X7	0.0039	0.0393	−0.0764	0.0978
X8	0.0326	0.0552	−0.0420	0.1786
X9	−0.0045	0.0390	−0.0949	0.0736

**Figure 1:**
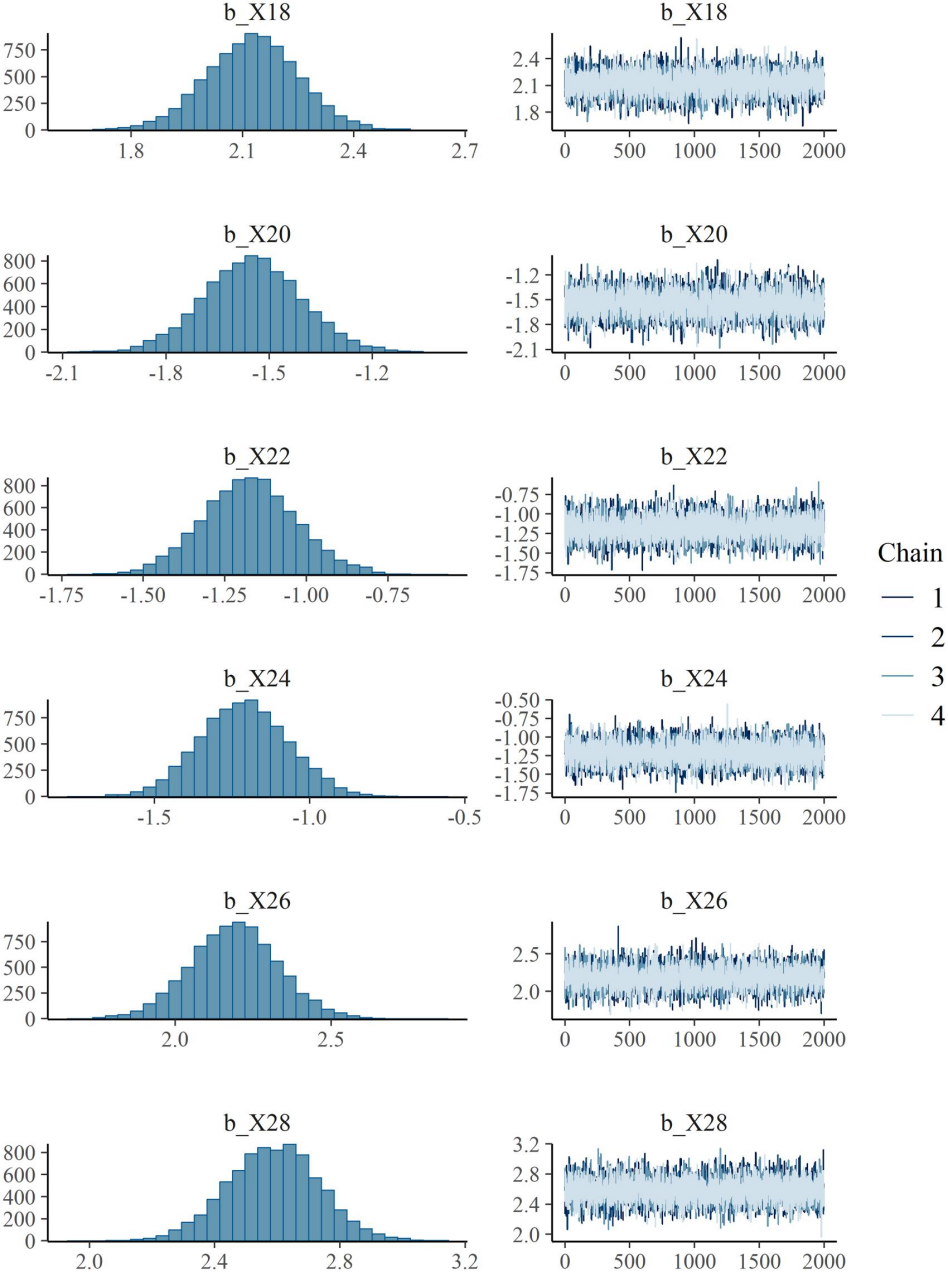
Density and trace plots of selected non-zero effects from the simulation.

We can also examine trace plots and density plots (Fig. 1) using the *plot* function for specific variables.

plot (fit, variable = c(“b_X18”, “b_X20”, “b_X22”, “b_X24”, “b_X26”, “b_X28”), nvariables = 6)

**Figure 2:**
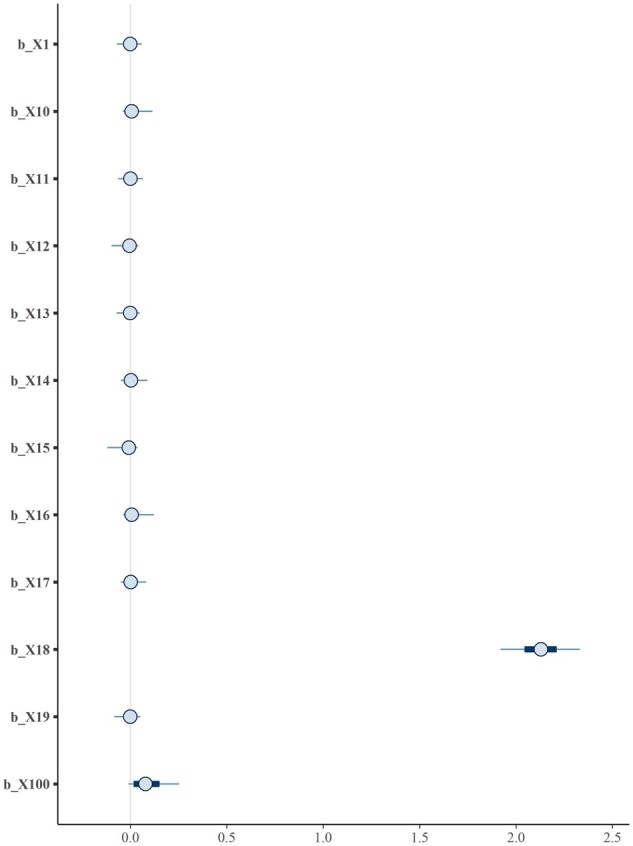
Posterior mean and 95% credible interval from MCMC draw for selected effects.

Additionally, the *mcmc_plot* function can be used to visualize posterior draws for selected parameters. For example, we can display the credible intervals for variables that start with X1 (Fig. 2).

mcmc_plot(fit, variable = “^b_X1”, regex = TRUE)

In this example, we incorporate phylogenetic relationships by including a similarity matrix. Users may also set the *similarity* argument to NULL to ignore the relationships between taxa. The function *sim_b* can be used in a similar manner to generate a binary response, and model fitting is achieved by setting the family argument to Bernoulli in the *bcglm* function.

The simulation utilities included in **BCGLMs** are simple demonstration tools, designed to illustrate the basic workflow of Bayesian compositional generalized linear models rather than providing a fully flexible or highly customizable simulation framework. Several parameters in these functions are intentionally fixed or limited to ensure reproducibility and ease of use. Users can leverage these examples to explore model fitting, posterior summaries, and diagnostic plots; however, the functions are not intended for extensive simulation studies or advanced parameter exploration. Users may extend these functions to explore additional simulation scenarios as needed.

## 5 Conclusions

We have developed a freely available R package, **BCGLMs**, to address the analytical challenges associated with compositional microbiome variables and different types of outcomes. Although built on the **brms** package, **BCGLMs** extends its functionality in several keyways to enhance usability, integration, and accessibility. First, it directly models covariates on the simplex, which is appropriate for microbiome relative abundance data. Second, it implements a structured version of the regularized horseshoe prior, enabling adaptive and interpretable shrinkage for high-dimensional compositional features. Third, **BCGLMs** allows the incorporation of phylogenetic relationships among taxa via prior specification, providing more biologically informed modeling. Together, these features offer a streamlined workflow for compositional microbiome data analysis, reducing the need for complex preprocessing or manual model specification. In addition, **BCGLMs** improves accessibility by packaging advanced Bayesian methods into a user-friendly interface, lowering the expertise required to apply these models in practice.

**BCGLMs** incorporate phylogenetic relationships using kernel-based similarity matrices, but its primary focus remains on directly modeling the compositional structure of microbiome data. In contrast to many kernel- or distance-based approaches, **BCGLMs** employs a fully Bayesian MCMC framework, providing posterior distributions for regression parameters rather than only point estimates (Zhang *et al.* 2024a,b). This allows for richer inference, including uncertainty quantification and interpretable shrinkage, which is particularly valuable when analyzing high-dimensional compositional features with complex dependencies.

Although primarily designed for microbiome studies, the Bayesian modeling framework underlying **BCGLMs** is broadly applicable to a wide range of compositional data contexts. With its extensible design and scalability, the package provides a versatile toolkit for researchers working with both standard and large-scale compositional datasets.

## Data Availability

The data underlying this article are available in GitHub - Li-Zhang28/BCGLMs.
